# Reconstruction of a thumb metacarpophalangeal bone defect using the Masquelet technique: A case report

**DOI:** 10.1016/j.ijscr.2025.111425

**Published:** 2025-05-13

**Authors:** Gauthier Lagarde, Omar Alawadhi, Olivier Camuzard, Thierry Balaguer, Brieuc Monin, Elise Lupon

**Affiliations:** a Department of Plastic and Reconstructive Surgery, Institut Universitaire Locomoteur et du Sport, Pasteur 2 Hospital, University Côte d'Azur, Nice, France; b Laboratory of Molecular PhysioMedicine (LP2M), UMR 7370, CNRS, University Côte d'Azur, Nice, France

**Keywords:** Induced membrane, Bone graft, Bone defect, Osteoarthritis, Thumb reconstruction, Case report

## Abstract

**Introduction:**

Bone defects of the thumb secondary to trauma or infection pose a significant challenge for surgeons. The limited therapeutic options in such cases may, in the most severe scenarios, necessitate amputation, which has devastating functional consequences. The induced membrane technique, described by Masquelet, has proven effective in reconstructing bone loss in the lower limbs, particularly in septic contexts. However, its application in hand surgery remains underreported.

**Case presentation:**

In this case, we describe a bone and joint defect of the thumb's metacarpophalangeal joint, complicated by osteoarthritis following a dog bite injury. After failure of initial osteosynthesis, resulting in bone and joint destruction, we performed a two-stage metacarpophalangeal arthrodesis using an iliac bone graft and Masquelet's technique.

**Discussion:**

The permanent use of a cement spacer is often employed in clinical practice, but it frequently leads to complications, including spacer fractures, pain, and dislocations. Our proposed method is simple, reproductible, and applicable in both emergency and non-emergency settings.

**Conclusion:**

Our findings suggest that the Masquelet technique represents a promising reconstructive option for managing bone loss in osteitis with a high risk of amputation. This approach enables the preservation of sufficient thumb length to maintain pollici-digital function, allowing for satisfactory gripping ability. Further long-term studies are necessary to confirm these preliminary results.

## Highlights

• The Masquelet technique can effectively preserve thumb function in severe osteoarticular injuries.
• Two-stage induced membrane procedures may avoid amputation in post-infectious bone loss.
• Conservative salvage should be considered prior to alternatives like amputation or toe transfer

## Graphical abstract

**Figure:**
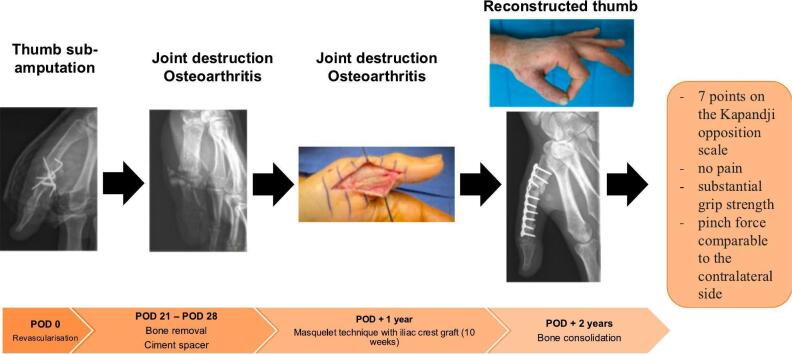

## 1 Introduction

The thumb contributes to approximately 40 % of hand function through its roles in opposition and circumduction, requiring a balance of mobility and stability [1]. Because of its essential function, the thumb is often the primary focus of reconstructive procedures following trauma [2]. In France, among the 1.4 million hand injuries reported annually, the thumb is involved in more than 25 % of cases [3].

Animal bites are a common source of osteo-articular infections, which can lead to bone destruction. These infections require appropriate antibiotic treatment, skeletal stabilization, and bone tissue supplementation. The hand is the second most common site of osteoarticular infection after the knee [4]. The amputation rate secondary to osteomyelitis of the tubular bones approaches 39% [5]. In cases of composite digital trauma involving multitissue lesions, bone stabilization is the first stage of treatment, preceding vascular and nervous repair. Restoring skeletal length is a major challenge in the presence of a significant bone defect. Alternatives to amputation are limited and include bone distraction, structural allografts, arthrodesis, or vascularized bone grafts [6]. In manual laborers who require strong grip function, skeletal robustness becomes a critical factor and must be carefully considered when planning the reconstruction.

The induced membrane technique, described by Masquelet, enhances reconstruction of lower limb bone defects in septic conditions by restoring both bone integrity and strength. More recently, its application to the hand has attracted growing interest as a promising option for managing osteitis with a high risk of amputation [7]. This technique can effectively preserve segmental length while maintaining satisfactory function. The Masquelet-induced membrane technique is a two-stage procedure. The first stage consists of the surgical debridement of necrotic tissue and the implantation of a cement spacer matching the dimensions of the bone loss. The second stage involves autologous bone grafting after the removal of the cement while preserving the induced membrane [8]. The pseudosynovial membrane secretes osteomodulating and angiogenic growth factors, such as VEGF, TGF-β, and BMP2, as studied by Pelissier et al [9]

In this report, we describe a case of metacarpophalangeal bone and joint loss of the thumb, complicated by osteomyelitis following a dog bite injury in a manual laborer. The condition was managed using an iliac crest autograft and a two-stage metacarpophalangeal arthrodesis based on the Masquelet induced membrane technique.

## 2 Case report

**Fig. 1:**
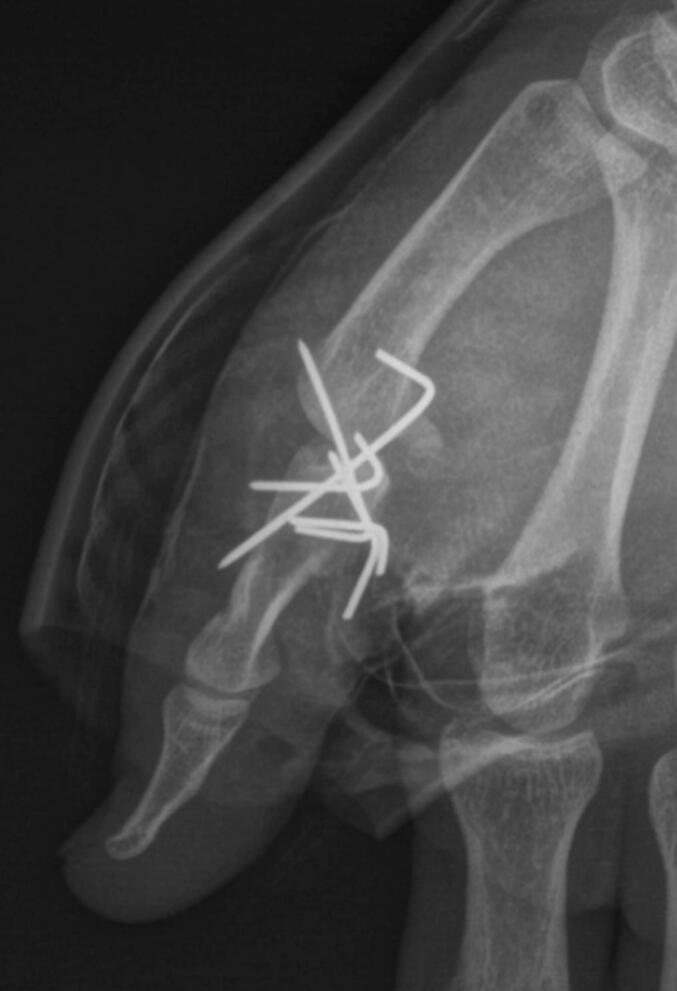
Lateral radiograph at the first operative stage during replantation.

A 31-year-old right-hand dominant man, working as a dog handler, with no significant medical history but an active smoking habit, presented to the emergency department with a dog bite injury to the left thumb. On initial examination, a major wound was observed in the palmar T2 zone, with dorsal attachment and signs of devascularization (sub-amputation). Radiographs revealed substantial bone and joint severe injury at the first phalanx, first metacarpal, and metacarpophalangeal (MCP) joint. The initial sensory examination showed normal sensation on the dorsal aspect of the thumb but no sensation on the volar aspect. The patient underwent urgent revascularization, debridement, open reduction, and temporary MCP arthrodesis using five small Kirschner wires. The arteries and nerves were anastomosed using non-absorbable 9.0 sutures, and intraoperative assessment confirmed patency of the anastomosis (Fig. 1). Immediately postoperatively, the finger showed signs of revascularization, and the initial postoperative course was satisfactory. Management included single-agent antiplatelet therapy for 45 days and a 10-day course of antibiotics. The patient was discharged after five days of inpatient monitoring and was followed up weekly.

At three weeks postoperatively, the patient presented with purulent wound discharge and radiographic signs of osteitis, although the thumb remained viable. At four weeks, despite the absence of bone consolidation, the decision was made to remove the devitalized bone and fixation material and to place a 3-centimeter antibiotic-impregnated polymethylmethacrylate (PMMA) spacer spanning the first phalanx. The patient was subsequently lost to follow-up.

**Fig. 2:**
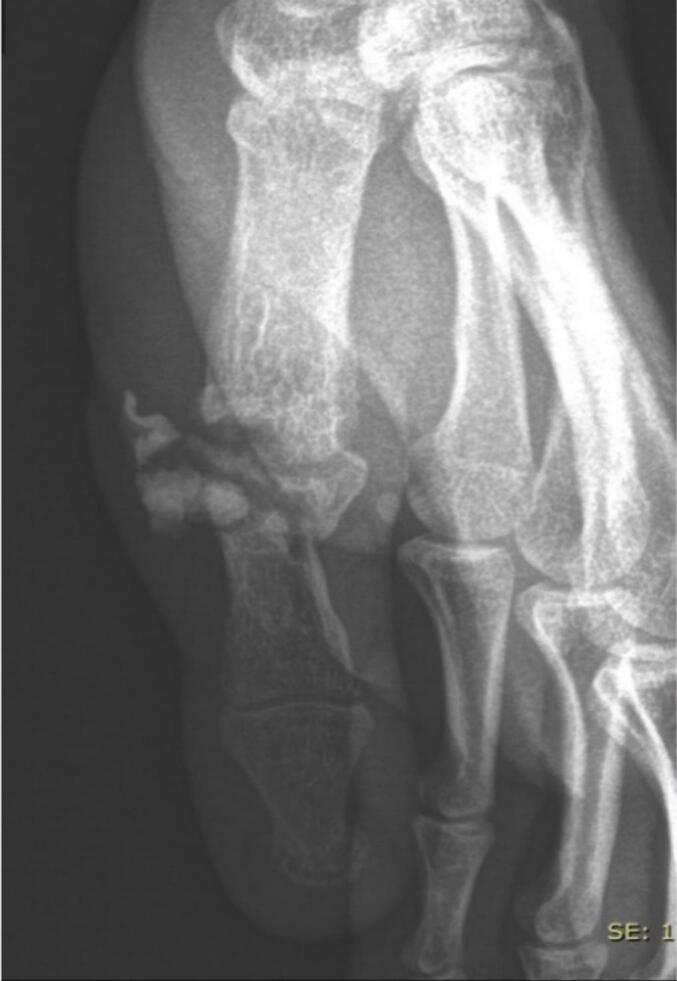
Frontal radiograph at 3 weeks showing osteoarthritis, necessitating the removal of the osteosynthesis material.

One year later, he returned with a palmar draining fistula with purulent discharge and exposed cement. He reported significant pain and total functional impairment. Radiographs confirmed complete joint destruction with chronic osteoarthritis (Fig. 2). A two-stage surgical treatment was planned using the Masquelet technique with antibiotic coverage. The first stage, performed one year after the initial trauma, involved bone resection with fistulectomy and placement of an antibiotic-loaded PMMA spacer for ten weeks. No osteosynthesis hardware was used to secure the spacer between surgical stages; immobilization was maintained with an orthosis. The fixation strength of the PMMA spacer was deemed sufficient to allow stabilization with orthotic support alone. Dual antibiotic therapy, including rifampicin and fluoroquinolone, was administered for ten weeks based on microbiological findings.

**Fig. 3:**
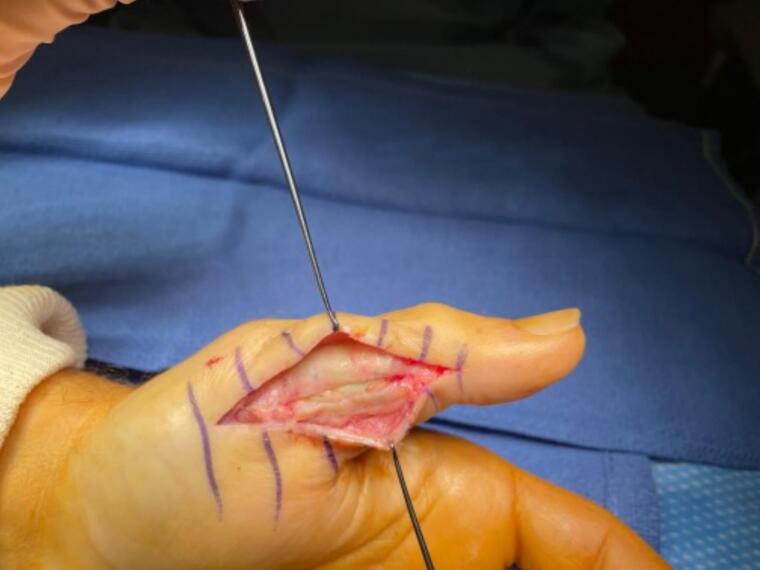
Photograph of the thumb showing the lateral opening of the Masquelet-induced membrane at one month, allowing for the replacement of the cement with iliac crest bone.

During the second surgical stage, the antibiotic spacer was carefully removed while preserving the induced membrane, which was approximately 2 mm thick and continuous (Fig. 3). A tricortical iliac crest autograft was harvested and designed to fill the defect, supplemented with allograft composite bone within the membrane. A long locking plate (2 mm) contoured to 15° of flexion was fixed with screws to achieve the definitive arthrodesis position. The induced membrane was sutured around the graft to optimize its integration.

Following surgery, the thumb remained viable, and intraoperative radiographs confirmed stable fixation. The patient was immobilized with a gauntlet splint for three months postoperatively, both due to non-compliance with postoperative recommendations (continued tobacco use, failure to maintain strict immobilization) and to maximize the chances of success in the context of multiple surgical revisions.

The patient underwent monthly radiographic follow-ups. At the first postoperative visit, ten days after surgery, radiographs showed stable fixation with no displacement, and the surgical wound was well-healed. Passive physiotherapy was initiated immediately after surgery, followed by active physiotherapy at three weeks. Weight-bearing activities were permitted at three months.

**Fig. 4:**
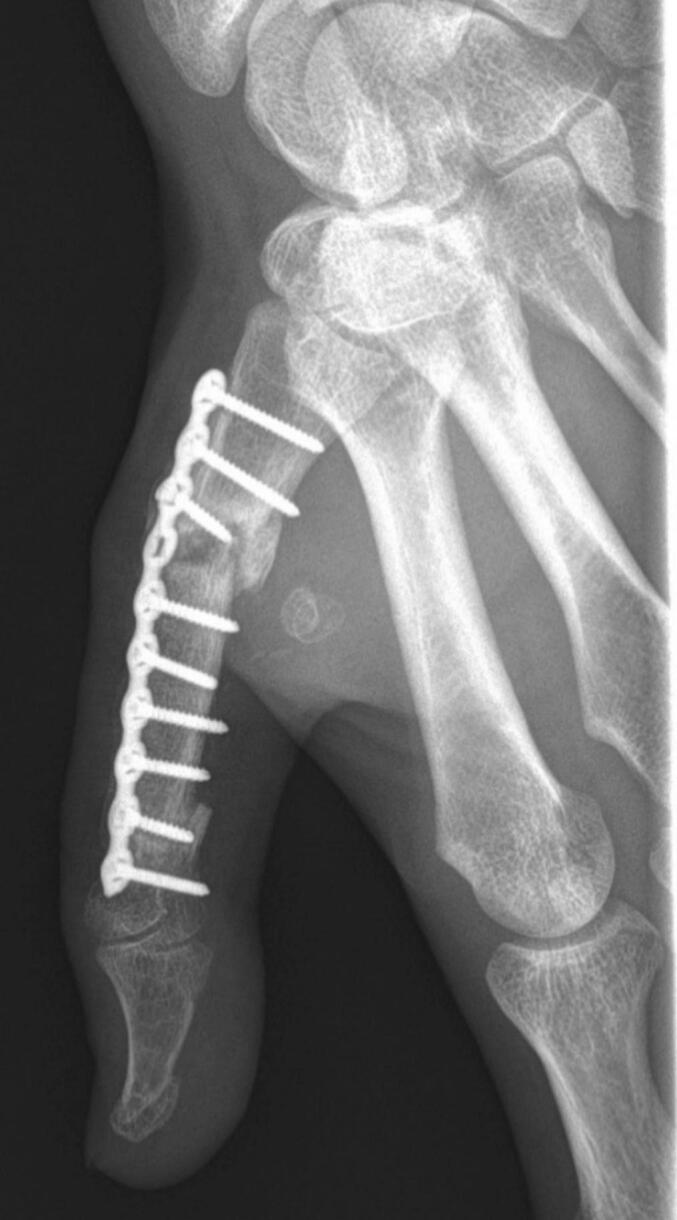
Frontal radiograph at 12 months postoperatively, demonstrating bone consolidation with ossification.

**Fig. 5:**
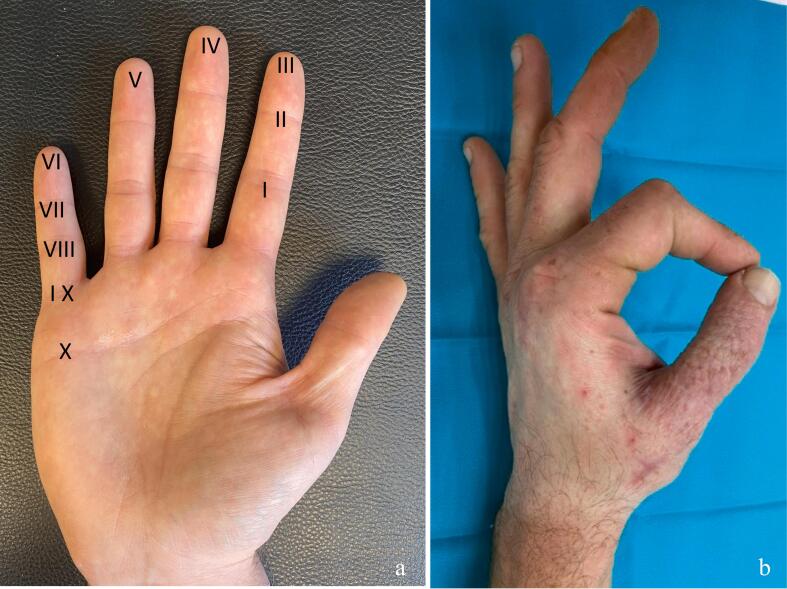
Kapandji opposition scale. a. Kapandji score for assessing the opposition of the thumb. b. Photograph of the patient thumb at 12 months postoperatively, demonstrating good mobility, achieving a score of 7 points on the Kapandji opposition scale.

At one year postoperatively, radiographs confirmed bone consolidation (Fig. 4). The thumb demonstrated good mobility, with a Kapandji opposition score of 7 (Fig. 5). Other thumb joints preserved normal range of motion, as mobility was maintained through the trapeziometacarpal and interphalangeal joints. Functionally, the patient reported no pain, with grip and pinch strength comparable to the contralateral side and to his pre-injury status. He returned to manual work and expressed full satisfaction with the functional outcome of the thumb. The scars were considered fully acceptable by the patient. Given the absence of complaints, it was decided not to remove the osteosynthesis hardware.

## 3 Discussion

The management of hand trauma—particularly thumb injuries complicated by chronic osteoarticular infection—remains a significant surgical challenge, often associated with a high risk of amputation. While permanent cement spacers are commonly used in clinical practice, their use is frequently associated with complications such as spacer fracture, pain, and dislocation. This report presents a non-compliant patient, which justified the use of a simple and efficient surgical approach—avoiding microsurgery, prolonged hospitalization, and allowing for straightforward physiotherapy. Report presents an original application of a well-established and reliable technique that facilitated the preservation and functional recovery of the patient's thumb.

In cases of post-traumatic or septic bone loss, the induced membrane technique offers a superior conservative approach [10,11]. This method is simple, reproducible, and applicable in both emergency and elective settings. It enables effective control of chronic infection through thorough debridement while initiating reconstruction and preserving skeletal length. Several studies, including those by Lum et al., support a conservative approach prior to considering amputation or more complex reconstructions such as toe-to-thumb transfers [12]. There is limited literature regarding the application of this technique to the hand and even fewer reports specific to the thumb [[12], [13], [14], [15]]. Our review of the literature on metacarpophalangeal trauma with bone defects treated using the Masquelet technique highlights a consensus among authors regarding the importance of prioritizing conservative management at the thumb level. A detailed summary of relevant studies is presented in Table 1. Most involve post-traumatic or infectious etiologies, with an interval between the two surgical stages ranging from 17 days to 7 months [[13], [14], [15]]. The need for additional procedures, such as skin flaps, remains rare and typically arises when soft tissue loss is present [14]. Reported bone consolidation was achieved in all cases, generally within 2 to 12 months [[12], [13], [14]]. These results, although based on small series or case reports, suggest that the Masquelet technique may be a reliable option for managing bone defects in the hand, even in complex scenarios. Although current outcomes are promising, further case series are required to validate these results and establish stronger evidence.

**Table 1:** Table summarizing the literature on metacarpophalangeal trauma with bone defects treated using the Masquelet technique.

Reference	Number of patients	Etiology of bone defect	Associated lesions	Delay between T1-T2 Masquelet's procedure	Additional surgery	Time to consolidation	Follow- up
Flammans [13]	11	3 sepsis, 8 trauma	No	17 days- 7 months	No	1,5–12 months	5 years
Lum [12]	1	Trauma	No	4 weeks	No	2 months	2 years
Lim [15]	1	Tumor	No	3 weeks	No	_	9 months
Luttenberger [14]	1	Trauma	Skin defect	6 weeks	Skin flap	8 weeks	8 months

In our case, the Masquelet technique successfully preserved thumb-to-finger function, offering a more functional and aesthetically acceptable result than amputation. Amputation at the thumb level is associated with significant disability and poor functional prognosis [16]. When a vascularized thumb with extensive bone loss is salvageable, it should be prioritized as the first-line treatment. Alternative reconstructive procedures—such as index finger pollicization or toe-to-hand transfer—may compensate for post-amputation deficits but should be reserved as last-resort options. The sacrifice of a potentially functional structure is only justified when all conservative options have been exhausted or deemed unfeasible. While toe transfers can restore function, patient acceptance is often limited due to the heterotopic appearance of the reconstruction and the morbidity associated with the donor site [17].

Several limitations should be acknowledged in this case. This report, following the SCARE guidelines [18], describes a single case of thumb reconstruction using this technique by a trained hand surgeon, and further case series are necessary to draw broader conclusions. Additionally, the postoperative follow-up period remains relatively short, highlighting the need for longer-term evaluation to confirm these promising preliminary results. A systematic assessment of the technique's indications, associated risks, and long-term outcomes is essential to better define its role in complex reconstructive procedures.

## 4 Conclusion

The application of the Masquelet technique to the hand represents a promising option for managing large bone defects in osteitis cases with a high risk of amputation. This technique allowed for the preservation of sufficient bone length to maintain pollici-digital function, enabling satisfactory gripping. Further studies are required to validate these initial findings over the long term.

## SCARE guideline

The work has been reported in line with the SCARE criteria 2023.

## Author contribution

GL: Gauthier Lagarde.

BM: Brieuc Monin.

O.M: Omar Alawadi.

T.B: Thierry Balaguer.

O·C: Olivier Camuzard.

E.L: Elise Lupon.

**G.L:** conceptualization, investigation, project administration, writing – original draft. **B.M:** conceptualization, investigation, methodology, writing – original draft. **O.M:** investigation, software, writing – original draft**. T.B and O·C**: supervision, writing – review and editing. **E.L:** project administration, supervision, writing – review and editing.

## Declaration of patient consent

Written informed consent was obtained from the patient for publication of this case report and accompanying images. A copy of the written consent is available for review by the Editor-in-Chief of this journal on request.

## Ethical approval

This study was performed in line with the principles of the Declaration of Helsinki. This study was approved by our institution's Research Ethics Committee.

## Guarantor

All authors in the article accept full responsibility for the work, have access to the patient's information, and decide to publish.

## Funding

The authors declare that no funds, grants, or other support were received during the preparation of this manuscript.

## Declaration of competing interest

The authors declare no conflicts of interest.

## Data Availability

The datasets of the present study are available from the corresponding author upon request.
